# Attention-LSTM based prediction model for aircraft 4-D trajectory

**DOI:** 10.1038/s41598-022-19794-1

**Published:** 2022-09-15

**Authors:** Peiyan Jia, Huiping Chen, Lei Zhang, Daojun Han

**Affiliations:** School of Computer and Information Engineering, Henan University, Kaifeng, China

**Keywords:** Aerospace engineering, Electrical and electronic engineering

## Abstract

Aviation activities are constantly increasing as a result of the growth of the global economic system. How to increase airspace capacity within the limited airspace resources while ensuring smooth and safe aircraft operations is a challenge for civil aviation today. Air traffic safety is supported by accurate trajectory prediction. The way-points are relatively sparse, and there are many uncertain factors in the flight, which greatly increases the difficulty of trajectory prediction. So, it is vital to enhance trajectory prediction accuracy. An attention-LSTM trajectory prediction model is proposed in this paper, which is split into two parts. The time-series features of the flight trajectory are extracted in the initial stage using the long-short-term memory neural network (LSTM). In the second part, the attention mechanism is employed to process the extracted sequence features. The impact of secondary elements is reduced while the influence of primary ones is increased according to the attention mechanism. We used the advanced models in trajectory prediction as the comparison models, such as LSTM, support vector machine (SVM), back propagation (BP) neural network, Hidden Markov Model (HMM), and convolutional long-term memory neural network (CNN-LSTM). The model we proposed is superior to the model above based on quantitative analysis and comparison.

## Introduction

With the rapidly growth of the civil aviation sector in recent years, air traffic flow has expanded dramatically, putting a strain on airspace resources. According to figures from the International Civil Aviation Organization, global air traffic flow doubles every seven years.The current air traffic and navigation system’s operating capacity is reaching saturation. Countries around the world have proposed various coping strategies to coordinate airspace resources, such as Single European Sky ATM Research (SESAR)^1^ in the United Kingdom and the Next Generation (NextGen)^2^ Transportation System in the United States, in response to increasingly serious problems such as limited airspace, flight delays^3^, and intensified conflicts. These two missions have aided the development of Automatic Dependent Surveillance Broadcast (ADS-B)^4^, a system that integrates modern technologies such as satellite navigation, communication technology, aerial equipment, and ground equipment. It is a significant technological breakthrough in the evolution of the aviation system. Solving air traffic route regulation and achieving optimum operational efficiency is also a significant technical achievement for the global civil aviation sector. ADS-B provides civil aviation with a safer and more efficient means of air traffic surveillance by collecting information and accurately positioning ground wireless sensor networks. This effectively improves the operational situational awareness of controllers and pilots, enhances the control capability of airlines, expands surveillance coverage, and improves air traffic safety, airspace capacity, and operational efficiency. As a result, determining how to employ ADS-B data analysis to enhance airspace efficiency, expand airspace capacity, improve flight safety, minimize flight delays, and achieve “low-carbon environmental protection” is a critical component of the civil aviation policy.

One of the current effective tactics based on restricted airspace resources is to minimize the minimum spacing of airplanes, thus improving air flow^5^ . The implementation of various countries’ plans to relieve airspace tension has led to the proposal of an air traffic management model based on 4-D trajectory operations (TBO), which is based on accurate aircraft 4-D trajectory prediction, sharing trajectory dynamic information among air traffic control, airlines, and aircraft, and realizing collaborative decision-making between flight and control. On the basis of longitude, latitude, altitude, and time, the 4-D trajectory data has been transformed and upgraded in hardware and software to achieve a more accurate and quick data transmission standard, which sets a solid foundation for accelerating the development of the civil aviation air traffic management system. Using the 4-D trajectory, the precision of the anticipated arrival time of the aircraft is enhanced from the minute level to the ten-second level, ensuring smooth and safe aircraft operation while boosting airflow. 4-D aircraft trajectory flight, which comprises longitude, latitude, altitude, and time, is a new trend in civil aviation and the major growth direction of civil aviation navigation technologies in China. The time series is added to the 3-D aircraft trajectory, and the aircraft is needed to reach the defined waypoint at the stated time, which is more favorable to air traffic flow management.

For various flight itineraries, the aircraft’s 4D trajectory information must be varied. The daily 4-D trajectory information for the scheduled trip, on the other hand, will fluctuate with changes in weather, payload, and cruising altitude. As a result, the 4-D trajectory’s specificity and dynamics may be utilized to evaluate and mine past trajectory data, as well as pre-calculate the waypoint when the aircraft arrives at the next instant. Real-time synchronization and updates across departments to ensure the aircraft’s safe and efficient operation based on collaborative decision-making.

The existing 4-D trajectory prediction accuracy is insufficient to fulfill the demands of civil aviation air traffic control. We need to figure out how to handle ADS-B data and use a more efficient temporal prediction model to increase aircraft trajectory prediction accuracy. As a result of the aforementioned issues, we apply the attention-LSTM model to predict aircraft trajectory data and preprocess the data to increase the efficacy of data training. The main contributions of this paper are as follows:

- An attention-LSTM model is proposed for the prediction of aircraft trajectory. On the basis of time series prediction, it pays more attention to the influencing factors between the data, further extracts the characteristics of the data, and uses the attention mechanism to strengthen the influence of special data, and attenuate the influence of unnecessary factors, which improves the prediction accuracy of aircraft 4-D trajectory. Compared with the current aircraft 4-D trajectory prediction, the prediction accuracy of the model we proposed is higher than other advanced models.
- Considering that different causes influence distinct phases of an aircraft’s trajectory, which is represented in historical aircraft trajectory data. As a result, in this experiment, not only the data from the 4-D aircraft trajectory is taken into account, but also the speed and deflection heading angle to improve data diversity and predictability.
- Use the sliding window, which helps to keep the anticipated trajectory regulated by the spatial span consistent. We pick the sliding window approach to choose the training data based on the properties of the aircraft trajectory data, which assures data continuity and is more favorable to model training.

The rest of this paper is organized as follows: the second part reviews the related research work on the current trajectory prediction; the third part elaborates on the principles and details of the prediction model proposed in this paper; the fourth part introduces the specific content of the experiment and shows that it outperforms other advanced models; and the last part summarizes and forecasts future directions.

## Related work

To accelerate the implementation of the aircraft 4-D trajectory-based air traffic management(4-D-TBO) project, the primary goal is to improve the prediction accuracy of aircraft trajectory^6^. Currently, most research on trajectory prediction is data-driven and relies on the data from ADS-B for analysis and processing. According to the structure and parameters of the algorithms, 4-D trajectory prediction methods are mainly classified into aircraft dynamic-based models, and flight state estimation methods and data-driven models based on machine learning. In recent years, machine learning methods have been continuously applied in various directions, such as natural language processing^7^, machine vision, edge computing^8^, image processing^9,10^ etc., and have achieved very good results. Therefore, they have been gradually applied in the direction of aircraft trajectory prediction^3^.

The main consideration in early air traffic control is prediction accuracy. Traffic controllers use the predicted trajectory to make corresponding emergency measures. There are mainly two methods: the aircraft-based dynamic model and the state estimation method. The method based on the aircraft dynamics model is to establish the kinematic equation with the forces in the process of the flight of an aircraft to predict the future trajectory. The state estimation method is based on the transformation of flight parameters of the aircraft in each state to build a state transfer model. Using such models requires in-depth knowledge of aircraft states, parameters, and flight intentions. Qiao et al.^11^ proposed a hidden markov model (HMM) trajectory prediction algorithm based on adaptive parameter selection, which adjusted parameters according to the dynamic changes in the movement process, as well as introduced a density-based trajectory division algorithm to improve the prediction efficiency. Liu and Li^12^ used aircraft intentions to guide the interactive multi-model algorithm for aircraft trajectory prediction and improve the accuracy of trajectory prediction by establishing a dynamic model based on the heading angle at the previous moment. Richard and David^13^ analyzed historical climb data around the world and studied 11 common aircraft types to improve the aircraft trajectory prediction accuracy by predicting some unknown point mass model parameters. These methods can learn data features from specific aspects and improve the accuracy of prediction, but there is no way to learn the relationship between the data adequately. On the other hand, the model has many parameters and the early research mainly considers the prediction accuracy of aircraft 3-D trajectory in real-time, so it cannot meet the needs of air traffic control in advance.

With the increasing in air traffic flow, the workload of controllers increases. How to make reasonable arrangements for air traffic in advance to ensure safe and orderly air traffic is a problem that needs to be solved at present. Air traffic management based on 4-D trajectory prediction adds time series to make predictions of various situations appearing in the airspace and helps controllers make decisions in advance. This method is considered the main means to reduce the controller load intensity problem. Shi et al.^14^ proposed an LSTM neural network model to link the long-term relationship with the current prediction task for aircraft trajectory prediction, which achieved good results in both 3-D and 4-D aircraft trajectory prediction. In order to further refine the model, Shi et al.^15^ also proposed a staged prediction model, which divided the aircraft flight process into three stages: climb, cruise, and descent, and proposed three constraints respectively to construct an LSTM neural network with embedded constraints. Ma et al.^16^ used a hybrid model of CNN and LSTM to extract spatiotemporal features in data, which improved the ability to learn data features to a certain extent. Considering that the historical aircraft trajectory data contains various influences such as wind speed, resistance, meteorology, etc., the influence weights of various factors need to be changed according to the transformation of the flight scenes. Therefore, we propose to use the attention mechanism for features weight learning.

The attention mechanism is favored by many researchers for its intuitiveness, versatility, and interpretability and is gradually being used to solve unique problems in different fields such as natural language processing, machine vision, multi-task learning, recommender systems, and graph systems. Galassi et al.^17^ proposed a unified attention architecture model to process text data from 4-D: input representation, distribution function, compatibility function, and input-output, and classify a large number of current works in the natural domain. Wang et al.^18^ introduced a series of attention models and RNN neural network applications in the field of machine vision and described in detail, the experimental results that show the superiority of attention-based neural networks in this field. With the continuous application of the attention mechanism, many researchers have started to use it in trajectory prediction tasks in recent years. Peng et al.^19^ proposed a SRA-LSTM model in which a social encoder uses the relative between pedestrians to obtain a representation of the social relationship between them, and later uses social interaction modeling to obtain the characteristics of social relationships between pedestrians. Tang et al.^20^ proposed an attention-based long short-term memory genetic algorithm (GA-LSTM), which combines spatiotemporal correlation analysis to predict urban road traffic flow. Messaoud et al.^21^ addressed a multi-head attention mechanism considering the joint representation of static scenes and agents to address multimodal future trajectory prediction. Lin et al.^22^ proposed a spatiotemporal attention long short-term memory neural network model (STA-LSTM) for vehicle trajectory prediction, which not only performs well in prediction performance but also has interpretability to explain the influence of historical trajectories and neighboring vehicles on the target vehicle. Based on this, in light of this, we proposed an attention-LSTM model that deeply integrates trajectory traits with model features to improve the accuracy of 4-D trajectory prediction. We will next go into great detail on the model’s overall structure and specifics.

## Model

### Attention-LSTM

**Figure 1:**
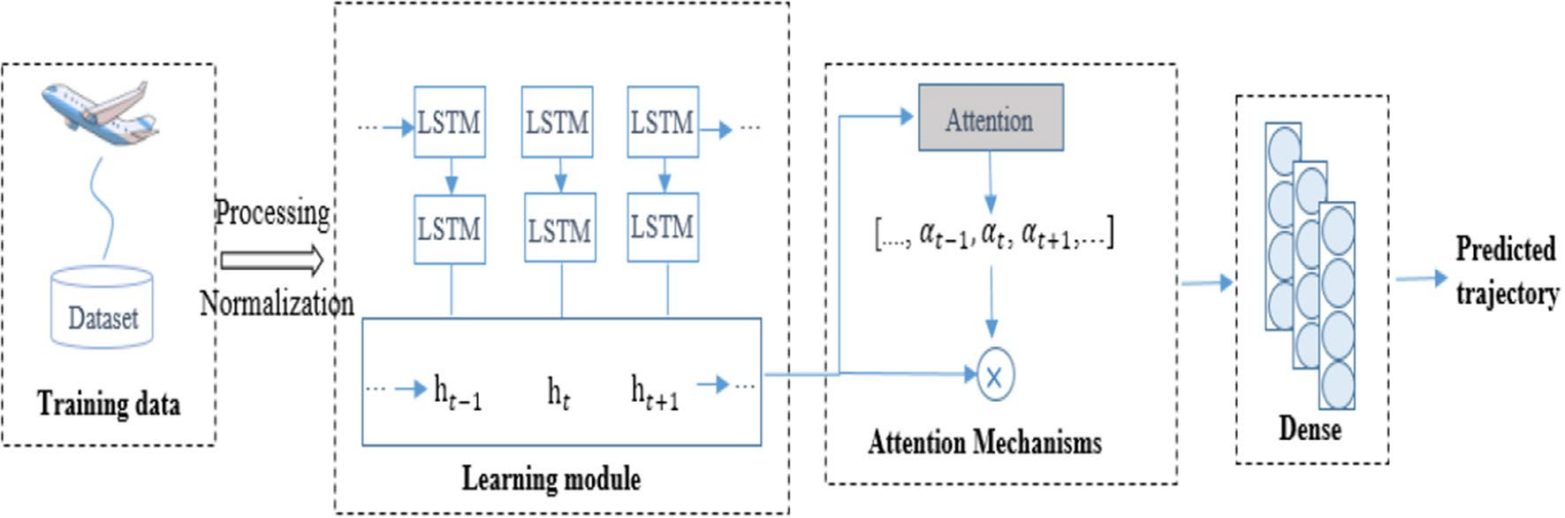
Attention-LSTM model.

The airplane trajectory points are sparser and the contributing elements are more complicated than ground traffic trajectories, resulting in low trajectory prediction accuracy. 4-D trajectory data is a typical time series, and the advantages of LSTM in processing time series may be leveraged to improve data interpretation and prediction. However, the flight path of the aircraft will change with changes in temperature, air pressure, and atmospheric density in different flight environments^23,24^, making a single LSTM model unable to accurately analyze the important influencing factors in the current flight state, resulting in a greatly reduced utilization of information data rate. This difficulty was satisfactorily solved by introducing the Attention mechanism. It can assign different attention to the model and improve the important factors for the model to automatically handle different situations. As a result, this research introduces a novel trajectory prediction model, the Attention-LSTM model. It makes advantage of the attention mechanism’s properties to pay greater attention to important influencing elements in prediction, increase the mining of tightly correlated influencing components, and improve prediction accuracy. The model architecture is shown in Fig. 1.

The model architecture proposed in this paper is separated into four modules, as indicated in the figure: data processing, prediction, attention mechanism, and fully connected layer. The data processing module is in charge of converting the original trajectory data into a format that the model can read directly; the prediction module is in charge of processing various input factors in order to obtain feature information; and the attention mechanism is in charge of learning a set of attention coefficients as well as the feature information. The fully connected layer gets the filtered feature information and analyzes it to provide the final prediction result. There are primarily two parts to these four modules. The time series features of the trajectory data are extracted using LSTM in the first step. In order to reduce prediction mistakes caused by secondary factors, enhance the impact of the primary factors, and increase prediction accuracy, the second part uses the attention mechanism to learn the features output by the first step.

### LSTM

Long sequence concerns have been solved due to the continuous developments in deep learning, with recurrent neural networks (RNNs) performing particularly well in this field. However, RNNs frequently experience disappearing or exploding gradients. In an attempt to improve the efficiency of deep learning networks, researchers are committed to improving the model’s convergence during the gradient descent process^25–28^. This issue is solved by the LSTM neural network proposal. The LSTM is a type of neural network made up of unit cells, each of which analyzes learning sequences using a specified gating mechanism, saves sequence features, and changes the current moment based on the input sequence’s characteristics. LSTM has a significant position in temporal sequence prediction^29^ and is now commonly employed in the field of trajectory prediction addition to its potential to solve the long-term dependence problem.

Unique to LSTM is the introduction of gating mechanisms: the input-gate, the output-gate, and the forget-gate. $x_t$ is the input at time t, $h_{(t-1)}$ is the output of the hidden layer at time t-1, and $h_t$ is the output at time t. The input-gate $i_t$ is the input inside the cell at time t and $W_i$ is the weight matrix. The data of $i_t$ is the tanh of weighting and biasing the output of $h_{(t-1)}$ and input of $x_t$. After the activation function is calculated, the value of $x_t$ is obtained.The specific calculation formula is as shown in Eq. (1).

1 $$\begin{aligned} i_t= \sigma (W_i*[h_{t-1},x_t ]+b_i) \end{aligned}$$

$W_o$ is the weight matrix of the output-gate, $o_t$ is the output at time t, which is calculated by the tanh of weighting and biasing $x_t$ and $h_{(t-1)}$,and finally update the input-gate by the activation function.The specific calculation formula is as shown in Eq. (2).

2 $$\begin{aligned} o_t= \sigma (W_o*[h_{t-1},x_t ]+b_o) \end{aligned}$$

In the forget-gate, $W_f$ is the weight matrix, the data of forget-gate $f_t$ is the tanh of weighting and biasing $x_t$ and $h_{(t-1)}$,and finally by the sigmoid activation function $\sigma$, the output value rangers between 0 and 1. The larger the value, the smaller the probability of being forgotten. When the value is 1, the input information $x_t$ is completely reserved. The specific calculation formula is as shown in Eq. (3).

3 $$\begin{aligned} f_t= \sigma (W_f*[h_{t-1},x_t ]+b_f) \end{aligned}$$

In the memory unit, $C_t$ is the state of memory cell at time t. The $f_t$ is multiplied by the $C_{(t-1)}$ and $i_t$ is multiplied by $\hat{C}_t$ ,before the two are summed to calculate $C_t$. The specific calculation formula is shown in Eq. (4). $W_C$ is weight matrix of the memory cell. The candidate cell state $\hat{C}_t$ is multiplied by the tanh of weighting and biasing $x_t$ and $h_{(t-1)}$. And then through the activation function, the $\hat{C}_t$ is obtained. The specific calculation formula is shown in Eq. (5).

4 $$\begin{aligned} C_t= & {} f_t* C_{t-1}+i_t*\hat{C}_t \end{aligned}$$

5 $$\begin{aligned} \hat{C}_t= & {} tanh(W_c*[h_{t-1},x_t ]+b_c) \end{aligned}$$

Finally, the output $C_t$ of the LSTM at time t is the product of the state of the memory cell $C_t$ after the tanh activation function and the output gate $o_t$ at time t. The specific calculation formula is as shown in Eq. (6).

6 $$\begin{aligned} h_t=o_t*tanh(C_t) \end{aligned}$$

### Attention

The attention mechanism is a signal processing mechanism discovered by researchers in the study of human vision in the 1990s. It is a special structure embedded in the study of machine learning models. It is mainly used to automatically learn and calculate input data pairs. The magnitude of the impact of the output data. Adding the attention mechanism to the deep learning model is equivalent to adding the thinking process of the human brain to the model, so that more valuable information can be paid attention to when processing information, and the information that has no effect on the task will be ignored, so it can be Improve forecast accuracy. The main weight parameters in the attention mechanism are $e_t$, $_t$ and $C_t$. Where $e_t$ is the weight score corresponding to different features at time t, the calculation formula is Eq. (7).

7 $$\begin{aligned} e_t= vtanh(W_e h_t+b_e) \end{aligned}$$

Among them, v and $W_e$ is the weight of the multilayer perceptron when calculating the attention weight, $b_e$ is the bias of the multilayer perceptron when calculates the attention weight, and $h_t$ is the output of the hidden layer at time t. $\alpha _t$ is the attention weight corresponding to different features at time t, and the calculation formula is Eq. (8).

8 $$\begin{aligned} \alpha _t= (expe_t)/(\Sigma ^n_{j=1} e_j ) \end{aligned}$$

Among them, $e_j$ is the weight scores corresponding to different features at time j. $C_t$ is the output of the entire attention mechanism at time t, and the calculation formula is equation (9).

9 $$\begin{aligned} C_t= \Sigma ^n_{j=1} \alpha _j h_j \end{aligned}$$

The attention mechanism is used to adaptively calculate and adjust the hidden layer state value corresponding to the original output feature, focus on important information, and fully learn and absorb it, highlighting important factors, and further pay attention to the influence of the predicted trajectory data, mining internal connections, Improve prediction accuracy.

## Experiment

**Figure 2:**
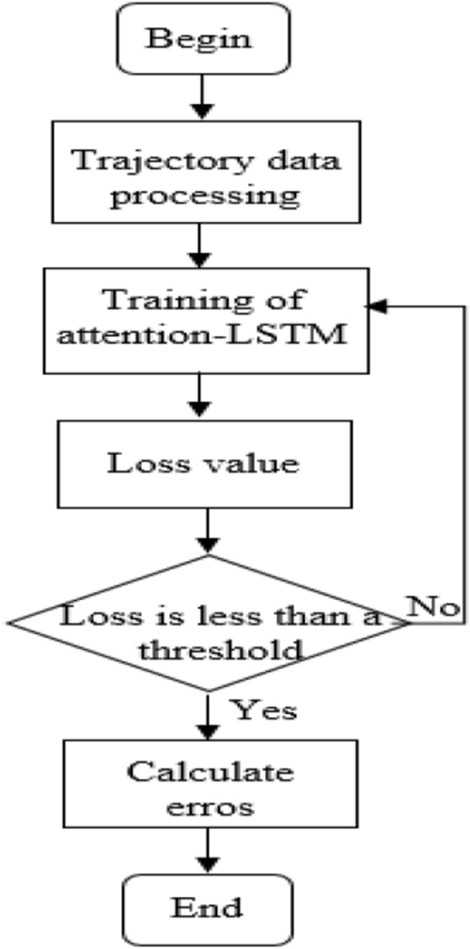
Flow chart of the experiment.

This part primarily describes the major aspects of the experiment, including data collection, assessment, the experimental environment, the settings for the comparative experiment and the ablation experiment, and experimental result analysis. The entire experimental process is shown in Fig. 2. We set up comparison experiments and used ablation experiments to evaluate each module’s effectiveness under quantitative conditions in order to validate the practicality of the attention-LSTM model proposed in this paper.

### Data

**Figure 3:**
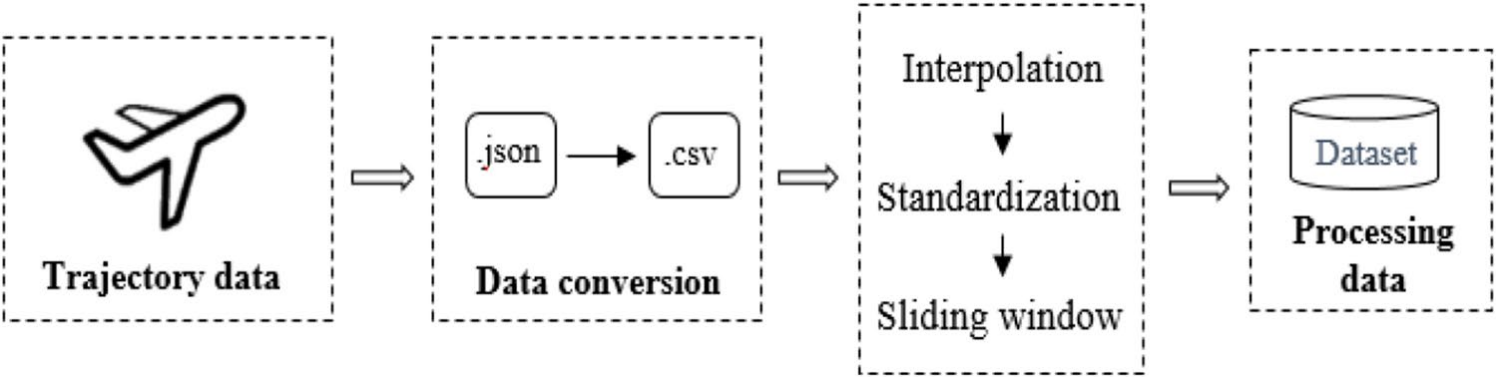
The acquisition of data set.

The ADS-B data from the Henan Air Traffic Management Branch of the Central and Southern Regional Administration of the Civil Aviation Administration of China in October 2020 is the source of the information used in this paper. Both static and dynamic data make up the data. The experiment makes use of dynamic data, including heading and speed, in addition to 4-D data (time stamp, longitude, latitude, and altitude). The time of data updates is 5 s. This paper utilizes flight data that lasts more than an hour for each flight to assure the adequate experimental data in this paper.We set a sliding window to modify the data, with a window size of 10 and a step size of 1, in order to better manage the spatial range of the input data and enhance the accuracy and smoothness of the forecast. The data is split into 55-s segments, where the first 50 s provide the historical data time range and the final 5 s serve as the predicted time range. This generates 1067 trajectories. We split these trajectories into training and test sets with a 7:3 ratio. The specific data set acquisition process is shown in Fig. 3.

### Evaluation

Typically, Euclidean distance is used to evaluate how similar two data sets are. Therefore, the primary evaluation criteria for the model in this paper are root mean square error (RMSE), mean absolute error (MAE), and mean relative error (MRE). Currently, the preferred evaluation methods in the field of 4-D trajectory prediction are these three error calculation values, which reflect the discrepancy between the actual flight trajectory and the predicted flight trajectory. Additionally, this paper chose dynamic time warping (DTW) to evaluate the similarity of sequences in order to confirm the dynamic change features of trajectory sequences.

### Experimental environment

The experiments in this paper are all implemented in the same computer configuration (CPU: Intel(R) Core (TM) i9-9900K, memory: 32GB, GPU: GeForce RTX 2080). All predictions are operated in the Python 3.7 environment, using the TensorFlow 2.1.0 GPU version as the framework.

### Experimental details

We set up several experiments to evaluate the effectiveness of the proposed method in this paper.

#### LSTM with different numbers of hidden units

We set up the model architecture through quantitative experiments on the hidden units of LSTM in order to prove that the prediction model chosen in this paper is the best model. We begin by setting out the LSTM’s hyperparameters. After previously training the model, we determined the best hyperparameters to be lr = 0.0001, batch size = 500, and dropout = 0.2. Then, to train the model, we fixed the LSTM’s layer number to 4 and selected the number of hidden unit neurons from a range of 50, 100, 200, and 400. The model has a better overall performance when the number of hidden unit neurons is set to 400, 200, 100, and 50, as shown in Table 1.

**Table 1:** The erros of different hidden units.

Hidden units	Erro	Longitude	Latitude	Altitude	Hidden units	Erro	Longitude	Latitude	Altitude
{50, 50, 50, 50}	RMSE	0.0511	0.1215	7.8101	{400, 50, 50, 50}	RMSE	0.0643	0.0948	7.8424
	MAE	0.0460	0.0859	6.9259		MAE	0.0567	0.0720	6.9761
	MRE (%)	0.0404	0.2805	0.0668		MRE (%)	0.0498	0.2278	0.0673
{100, 50, 50, 50}	RMSE	0.0573	0.0781	8.0129	{400, 100, 50, 50}	RMSE	0.0483	0.0638	8.9382
	MAE	0.0527	0.0639	7.1344		MAE	**0.0337**	0.0516	8.1498
	MRE (%)	0.0463	0.2070	0.0688		MRE (%)	**0.0297**	0.1640	0.0786
{100, 100, 50, 50}	RMSE	0.0599	0.0736	8.0127	{400, 100, 100, 50}	RMSE	0.0609	0.0663	7.3076
	MAE	0.0528	0.0620	7.1309		MAE	0.0548	0.0528	6.3989
	MRE (%)	0.0464	0.1978	0.0688		MRE (%)	0.0482	0.1673	0.0617
{100, 100, 100, 50}	RMSE	0.0555	0955	7.8217	{400, 100, 100, 100}	RMSE	0.0615	0.0576	8.3794
	MAE	0.0511	0.0867	6.8515		MAE	0.0559	0.0467	7.4772
	MRE (%)	0.450	0.2771	0.0661		MRE (%)	0.0491	0.1486	0.0722
{100, 100, 100, 100}	RMSE	0.0638	0.0601	8.3485	{400, 200, 50, 50}	RMSE	0.0499	0.1180	7.6607
	MAE	0.0594	0.0479	7.4050		MAE	0.0461	0.1044	6.7804
	MRE (%)	0.0522	0.1518	0.0714		MRE (%)	0.0405	0.3372	0.0654
{200, 50, 50, 50}	RMSE	0.0859	0.0893	8.6174	{400, 200, 100, 50}	RMSE	0.0513	0.0505	**5.1075**
	MAE	0.0751	0.0778	7.8161		MAE	0.0415	0.0394	**4.1833**
	MRE (%)	0.0660	0.2487	0.0754		MRE (%)	0.0365	**0.1250**	**0.0404**
{200, 100, 50, 50}	RMSE	0.0826	0.0862	8.2755	{400, 200, 100, 100}	RMSE	0.0652	0.1021	8.7724
	MAE	0.0757	0.0755	7.3301		MAE	0.0595	0.0821	7.8578
	MRE (%)	0.0665	0.2409	0.0708		MRE (%)	0.0523	0.2592	0.0758
{200, 100, 100, 50}	RMSE	**0.0411**	0.0641	7.5917	{400, 200, 200, 100}	RMSE	0.0580	0.1633	8.3036
	MAE	0.0378	0.0550	6.6656		MAE	0.0526	0.1395	7.4128
	MRE (%)	0.0332	0.1756	0.0643		MRE (%)	0.0462	0.4431	0.0715
{200, 100, 100, 100}	RMSE	0.0807	0.0536	8.3810	{400, 200, 200, 200}	RMSE	0.0488	0.1294	7.9396
	MAE	0.0719	0.0428	7.5210		MAE	0.0410	0.1105	7.0186
	MRE (%)	0.0631	0.1362	0.0726		MRE (%)	0.0360	0.3518	0.0677
{200, 200, 100, 100}	RMSE	0.0539	**0.0465**	8.4559	{400, 400, 200, 200}	RMSE	0.0754	0.0966	8.7700
	MAE	0.0479	**0.0386**	7.5092		MAE	0.0687	0.0802	7.8655
	MRE (%)	0.0421	0.1250	0.0724		MRE (%)	0.0604	0.2551	0.0759
{200, 200, 200, 100}	RMSE	0.0774	0.0995	8.0743	{400, 400, 400, 200}	RMSE	0.0698	0.1429	7.3242
	MAE	0.0700	0.0823	7.1947		MAE	0.0633	0.1249	6.3333
	MRE (%)	0.0615	0.2614	0.0694		MRE (%)	0.0557	0.4038	0.0611
{200, 200, 200, 200}	RMSE	0.0576	0.1098	8.5857	{400, 400, 400, 400}	RMSE	0.0645	0.1377	8.6786
	MAE	0.0549	0.0891	7.8214		MAE	0.0586	0.1151	7.7028
	MRE (%)	0.0482	0.2824	0.0755		MRE (%)	0.0515	0.3657	0.0743

Significant values are in bold

The parameters of LSTM are described in Table  2.

**Table 2:** Structure of LSTM.

	Layer	Size
1	LSTM	400
2	Dropout(rate = 0.2)	–
3	LSTM	200
4	Dropout(rate = 0.2)	–
5	LSTM	100
6	Dropout(rate = 0.2)	–
7	LSTM	50
8	Dropout(rate = 0.2)	–

#### Baselines

We chose the baseline model, which is currently pretty advanced in the field of trajectory prediction, and the modified models proposed by other researchers in recent years for comparison in order to further indicate the advanced nature of the model proposed in this paper. The following are the primary comparison experiment models used for this paper:

- SVM: SVM is frequently used in these areas because it performs binary classification and linear regression analysis on data using supervised learning.
- HMM: The Markov model is a random model in probability theory. It is a quantitative predictive model that is inspired by statistics and may be used to anticipate a dynamic forecasting technology of different data distributions at equal time intervals. Using a Markov model, data that predicts future time depends only on the current state. The Markov model is currently a common tool for predictive modeling and probabilistic forecasting.
- BP: BP is a multi-layer feedforward neural network trained by error back-propagation, and it is also one of the most widely used neural network models.
- CNN-LSTM: The model uses CNN and LSTM to extract spatial and temporal features of the trajectory data, respectively.

**Table 3:** The comparison of different models.

Models	Erro	Longitude	Latitude	Altitude
BP	RMSE	0.0496	0.5418	10.7567
	MAE	**0.0362**	0.7572	9.3835
	MRE (%)	**0.0318**	0.4373	0.0906
SVM	RMSE	0.1355	4.0218	95.8205
	MAE	0.1306	3.6250	85.2828
	MRE (%)	0.1149	10.3504	0.8318
HMM	RMSE	0.2018	1.0472	27.9649
	MAE	0.1625	0.8625	22.6675
	MRE (%)	0.1430	2.7301	0.2187
CNN-LSTM	RMSE	0.2076	4.0324	137.7852
	MAE	0.1753	3.9026	135.7427
	MRE (%)	0.1543	11.1639	1.3292
Attention-LSTM	RMSE	**0.0494**	**0.0464**	**3.9228**
	MAE	0.0397	**0.0373**	**2.9402**
	MRE (%)	0.0349	**0.1209**	**0.0284**

Significant values are in bold

As shown by Table 3, the comparison of the error results shows that our proposed attention-LSTM predicts more accurately than that of other models when analyzed using the evaluation indicators RMSE, MAE, or MRE, indicating that the model proposed in this paper is suitable for trajectory prediction. SVM is a traditional machine learning model, and while it is quite accurate, it still falls short of the deep learning model in some respects. This demonstrates that deep learning models are superior to machine learning models in the region of feature learning. The quantitative comparative experiments show that the attention-LSTM prediction results of the paper are better than those of the comparison model in terms of latitude and altitude, whereas the results of the prediction errors for longitude MAE and MRE are inferior to those of the BP neural network. In the latter subsection, we will plot the experimental predicted trajectory data and add a new evaluation standard, DTW, in order to further assess the two models’ accuracy.

#### Ablation study

The 4-D trajectory prediction model presented in this paper consists mostly of two modules. We set up two variant models for studying: Attention-LSTM without LSTM and Attention-LSTM without attention, in order to evaluate each module’s efficacy for trajectory prediction. We use RMSE, MAE, and MRE to analyze predicted trajectory points and actual flight data in order to generate quantitative measures. The effectiveness of each module of the model architecture proposed in this paper is illustrated by the Table 4, which demonstrates that eliminating any module from the Attention-LSTM will result in an increase in the error values of each item.

**Table 4:** The comparison of different ablation experiments.

Models	Erro	Longitude	Latitude	Altitude
Attention-LSTM w/o LSTM	RMSE	0.8308	3.4048	358.9445
	MAE	0.8029	0.2602	358.1155
	MRE (%)	0.7112	9.4773	3.5848
Attention-LSTM w/o Attention	RMSE	0.0513	0.0505	5.1075
	MAE	0.0415	0.0394	4.1833
	MRE (%)	0.0365	0.1250	0.0404
Attention-LSTM	RMSE	**0.0494**	**0.0464**	**3.9228**
	MAE	**0.0397**	**0.0373**	**2.9402**
	MRE (%)	**0.0349**	**0.1209**	**0.0284**

Significant values are in bold

### Analysis of results

**Figure 4:**
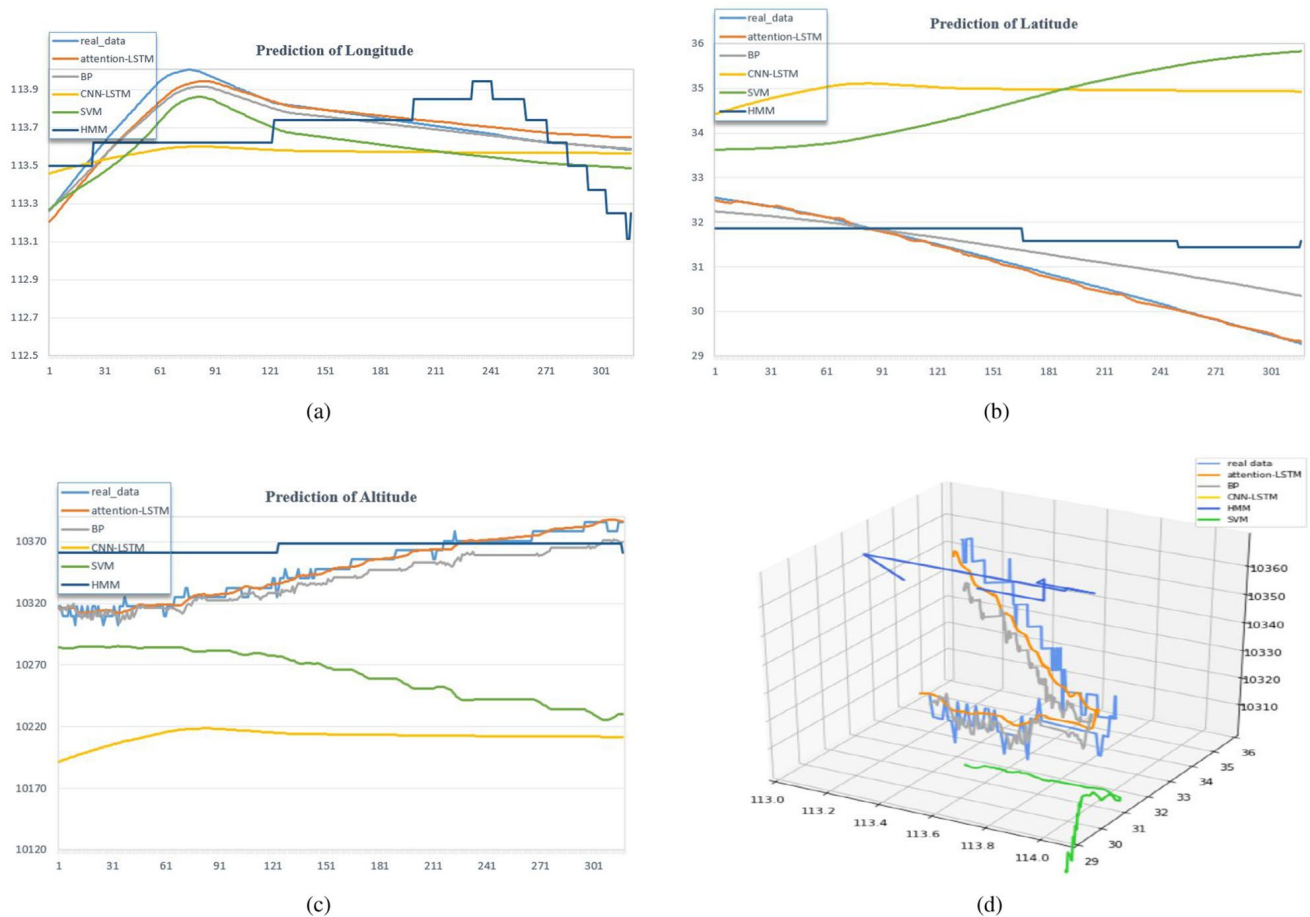
Prediction results of attention-LSTM, BP, CNN-LSTM, HMM, and SVM models.

Predicting motion trends and measuring how well the predicted trajectory matches real trajectory data are two ways to evaluate trajectory prediction accuracy. We use two-dimensional line graphs to show the prediction data of longitude, latitude, and altitude of different models in order to more clearly represent the results of the comparison experiments, as shown in Fig. 4a–c. Additionally, we combine the data from Fig. 4a–c in Fig. 4d to demonstrate the flight direction and trend in 3D. As shown in Fig. 4a–c, the predicted trajectory distributions of the HMM, BP, CNN-LSTM, and attention-LSTM models suggested in this paper can effectively represent the trend of real trajectories, in contrast to SVM solely. SVM does a good job of predicting longitude trends but fails to illustrate the latitude and altitude movement trends of the real trajectory. The HMM is a well-known approach in the field of trajectory prediction. The prediction results diverge significantly from the real data, despite being similar to the motion trend. This could be as a result of the enormous amounts of prediction data in this study, which caused frustrating prediction results. The complexity of the experimental data is a reason for the error that’s too high, even though the CNN-LSTM prediction result is smoother than the HMM’s. The advantages of the convolution module in the CNN-LSTM model can be effectively utilized and the accuracy of model prediction improved by enriching trajectory data and increasing data dimensionality, especially by adding complex scenes. On the dataset in this paper, both BP and attention-LSTM have good prediction results that are not only compatible with the movement trend of the real trajectory but also have a minimal difference between the prediction results and the real data.

**Figure 5:**
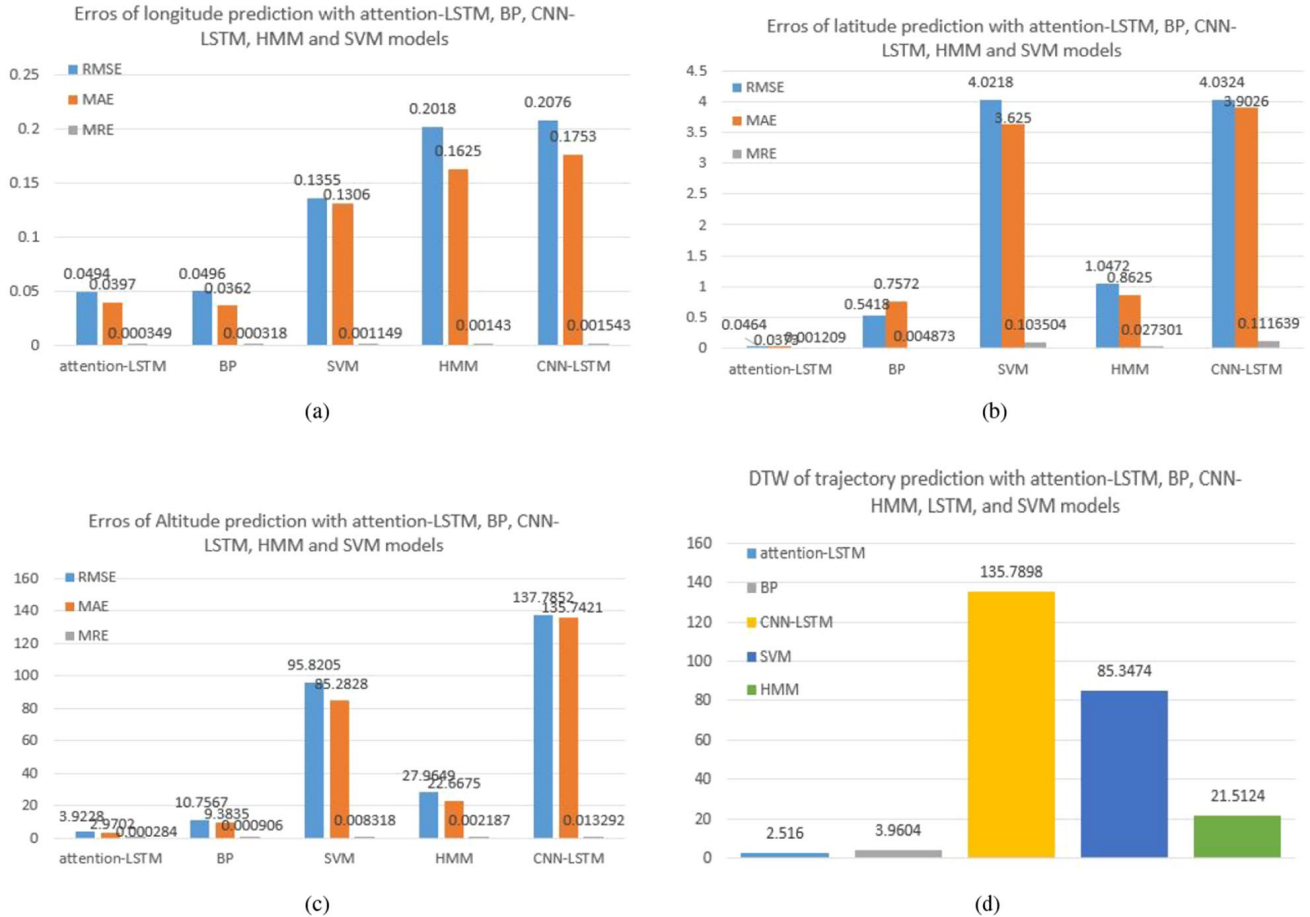
Evaluations of trajectory prediction with attention-LSTM, BP, CNN-LSTM, HMM, and SVM models.

We create bar charts of the RMSE, MAE, and MRE from the perspectives of longitude, latitude, and height to more clearly illustrate the error value between the trajectory prediction data and the real data between the comparison model and the proposed model, as shown in Fig. 5a–c. Moreover, we calculated the DTW values of the SVM, HMM, BP, CNN-LSTM, and attention-LSTM prediction trajectories and real data because DTW is commonly used as a metric for time series data to compare the similarity of two time series prediction trajectories. A bar graph was also designed, shown in Fig. 5d. The predicted trajectory of the model presented in this paper is more similar to real data, as shown in Fig. 5d.

## Conclusion

In order to better analyze and process ADS-B data, improve the accuracy of 4-D aircraft trajectory prediction, and realize the operation of intelligent air traffic control as soon as possible, this paper proposes an attention-LSTM 4-D aircraft trajectory prediction model. By adding an attention mechanism, the model pays more attention to the interaction between data on the basis of LSTM prediction, integrates valuable influence information, and improves the accuracy of prediction. In addition, a series of preprocessing on the ADS-B data used in the experiment is also a necessary means to improve the prediction accuracy in this paper. The attention-LSTM model proposed in this paper is compared with LSTM neural network, SVM, BP neural network, Hidden Markov Model (HMM) and CNN-LSTM neural network. Under the same experimental environment, the model architecture proposed in this paper outperforms the typical algorithms and most commonly used prediction models used in the trajectory prediction field. We also held ablation experiments to prove the efficiency of each module of the method in this paper. In the next step of research, we plan to consider more factors that affect the flight process, such as meteorology, geographic features, and the interaction between aircraft, etc., and improve our prediction model to adapt to the needs of emergencies.

## Data Availability

The data that support the findings of this paper are available from the Civil Aviation Administration of China Central and Southern Regional Administration but restrictions apply to the availability of these data, which were used under license for the current study, and so are not publicly available. Data are however available from the authors upon reasonable request and with permission of the Civil Aviation Administration of China Central and Southern Regional Administration.
